# An investigation into the player development pathway among youth soccer players in South Africa

**DOI:** 10.17159/2078-516X/2025/v37i1a20183

**Published:** 2025-08-15

**Authors:** W Steenbok, H Morris-Eyton, A Kubayi

**Affiliations:** 1 Department of Sport and Movement Studies, Faculty of Health Science, University of Johannesburg, South Africa; 2 Department of Sport, Rehabilitation and Dental Sciences, Tshwane University of Technology, Pretoria, South Africa

**Keywords:** coaching, talent, development, soccer

## Abstract

**Background:**

Scant research has examined soccer player development trajectories in South Africa. Therefore, research on South African talent development may help create environments conducive to players’ learning and growth.

**Objectives:**

The study aimed to investigate the factors affecting player development based on playing level (community team versus school academy) and playing phases (Youth Development Phase [YDP] versus Professional Development Phase [PDP]).

**Methods:**

The study adopted a cross-sectional research design. A total of 112 male soccer players (M_age_=16.2±1.2 years; 51 community-based team players and 61 school academy players; 73 YDP players and 39 PDP players) participated in the study. Data were collected using a 30-item Player Development Soccer Scale.

**Results:**

Results showed that coaching (4.27±0.75 arbitrary units (AU)), personal mindset (4.24±0.63 AU) and social support network (4.17±0.63 AU) were perceived as the most important factors contributing to player development. The least important factor was sport psychology (3.62±0.83 AU). However, compared to school academy players, community team players recorded significantly (*p<*0.01) higher scores on all the factors of the player development pathway. None of the player development factors showed a significant (*p*>0.05) difference between YDP and PDP.

**Conclusion:**

The current results may help teams support players psychologically so they can advance along their developmental pathway.

Soccer academies play a crucial role in the development of youth players.^[1]^ To ensure high standards in these academies, many developed nations, such as England, have introduced the Elite Player Performance Plan (EPPP) to increase the effectiveness of a player’s development trajectory. The EPPP divides athletic development into three phases based on age. These include the 1) Foundation Phase (U9–U11), 2) Youth Development Phase (U12–U16) and 3) Professional Development Phase (U17–U23).^[2]^ This categorisation is justified by Bloom,^[3]^ who distinguished three phases of talent development: the early, middle and later years.^[2]^ Similarly, Côté^[4]^ divided players into three phases: the sampling years (6–12 years old), the specialising years (13–15 years old) and the investment years (16 years and above).

Whilst EPPP outlines essential phases of talent development,^[2]^ South Africa lacks a standard player development pathway, as soccer teams rely on regional, provincial and national amateur schools and professional leagues to develop youth soccer players.^[1]^ According to the South African Football Association Academy Regulations,^[5]^ schools, amateur clubs and associate members operating within its youth soccer system must conform to ethical and appropriate development standards that include players’ care, coaching, education and a holistic long-term approach. The soccer academies are further encouraged to develop young players’ competencies and abilities through a sustained technical curriculum.^[6]^ To fulfil their development capacities, players need to be afforded the appropriate environment for transition from amateur to professional soccer.^[7]^ Several factors that are thought to impact this transition include coaching, sports science and performance analysis, social support networks, personal mindset, and sports psychology.^[8]^

For coaching, Harwood et al.^[9]^ have argued that positive coaching produces the best results in players’ early academy years since their behaviours and actions are easily shaped at this young age. At the same time, Lagestad et al.^[10]^ revealed that players benefit the most from coaching at a later age since it enables them to customise their in-game performance, having already acquired a wealth of technical knowledge from their academy experience.

Sports science and performance analysis play a crucial role in the current soccer ecosystem by improving individual and team performances, enabling players to better understand the game. When correctly applied, performance analysis can have a positive impact on youth development by helping players increase their awareness of how to perform in realistic game scenarios.^[8]^

The role of social support networks (friends, coaches and parents) and personal mindset has also been well documented.^[8]^ Personal mindset emphasises the benefits of reflecting on and assessing one’s own performance as a means of advancing self-development. As Finn and McKenna^[11]^ have argued, players must have a strong personal mindset and coping mechanisms to support them throughout difficult times so they can manage a range of events and potential pressures.

Lastly, sport psychology has been shown to impact talent development. In elite sports environments, all participants require a minimal degree of fulfilment of need to feel as though they belong.^[12]^ This is supported by Mills et al.^[13]^, who demonstrated that in elite youth development, individual psychological traits catalyse the growth of young athletes. These traits include self-awareness, resilience and goal-drivenness.^[ 13]^ As Cooper^[8]^ confirmed, a player’s psychological condition can significantly impact or interfere with the development of their skill, and identifying a player’s mental health problems (a task best performed by a sport psychologist) can mitigate negative consequences and guarantee that the player’s growth continues through the academy system.

Despite research examining player development pathways in soccer,^[8,13]^ there is little investigation within the South African context. This study aims to fill this gap by investigating the factors affecting youth player development in South Africa related to playing level—community team versus school academy—and phases of development—Youth Development Phase (YDP) versus Professional Development Phase (PDP). To facilitate and increase the number of talented players, Cooper^[8]^ has suggested enquiring into the differences among players in the different phases, particularly regarding their perspectives on their experiences. It is envisaged that the findings will lead to establishing a specially designed, tailored programme to facilitate players’ transition from amateur to professional soccer in South Africa.

## Methods

### Study design and sample

This study adopted a cross-sectional research design. A convenience sample of 112 male soccer players (Mean age=16.2±1.2 years; 51 community-based team players and 61 school academy players; 73 YDP players and 39 PDP players) participated voluntarily in the study.

### Research instrument

A 30-item Player Development Soccer Scale (PDSS) developed by Cooper^[8]^ was used to examine player development. The PDSS comprises five subscales: coaching (6 items), sport science/performance analysis (6 items), sport psychology (6 items), social support network (6 items) and personal mindset (6 items). Each item was scored on a five-point Likert scale ranging from 1 (strongly disagree) to 5 (strongly agree). The Cronbach alpha coefficient value for PDSS was 0.918.

### Data collection

This study received ethical clearance from the research ethics committee of the University of Johannesburg (REC1851-2022). Parents were asked for written informed consent before data collection, and the players had to sign an assent form to be eligible to participate in the study. Participants were made aware that this study was completely voluntary and that they might leave without incurring any penalty. Participants were assured that their answers would remain anonymous and that confidentiality would be maintained throughout the study. Two team managers with training as research assistants managed the data collection process and explained how to complete the questionnaire to the participants. The players received the questionnaires before or after their teams’ training sessions. Every participant was instructed to fill out the PDSS on their own. The questionnaire took approximately eight minutes to complete.

### Data analysis

The descriptive statistics (means and standard deviations, frequency counts and percentages) were used to analyse data. Furthermore, an independent *t*-test was computed to assess differences in the player development pathway based on type of team (i.e. community team vs school academy) and age group (i.e. YDP vs PDP). Cohen’s *d* effect size (ES) was performed to examine the meaningfulness of the difference and interpreted as follows: <0.2 (trivial), 0.20 (small), 0.50 (moderate) and 0.80 (large).^[14]^ The Cronbach alpha coefficient was used to examine the reliability of the PDSS. The significance level was set at *p*≤0.05, and all statistical analyses were computed using SPSS software version 28.

## Results

Table 1 shows the player development pathway scores for community team and school academy players. Overall, coaching (4.27±0.75 arbitrary units (AU), ES=0.75, moderate effect), personal mindset (4.24±0.63 AU, ES=1.24, large effect) and social support network (4.17±0.63 AU, ES=0.58, moderate effect) were perceived as the most important factors contributing to player development. The least important factor was sport psychology (3.62±0.83, ES=0.92, large effect). Compared to school academy players, community team players gave significantly (*p<*0.01) higher rankings to all the factors of the player development pathway.

As Table 2 shows, players in the YDP perceived that coaching (4.32±0.70 AU, ES=0.18, trivial effect) and personal mindset (4.26±0.61, ES=0.12, trivial effect) were the most important factors contributing to player development, whereas players in the PDP ranked social support network (4.20±0.56 AU, ES=0.06, trivial effect) as most important. None of the player development factors showed a significant (*p*>0.05) difference between the two groups.

## Discussion

This study aimed to investigate the player development pathway among youth soccer players in South Africa. The findings demonstrate that coaching is the most important factor influencing player development. This result supports Cooper ^[8]^ who demonstrated that strengthening coaching is the most important strategy for facilitating growth among youth soccer players. In this study, players on community-based teams had a significantly higher mean than players on academy teams, suggesting that academy coaches may be committed to creating and executing training programmes that accelerate players’ development pathways to increase their chances of being recruited by professional soccer academies. Furthermore, although not significant, coaching was shown to be the primary contributing factor impacting player development in YDP rather than PDP. Research by Lagestad et al.^[10]^ has shown that players benefit more from coach feedback than from hands-on coaching when they are older. These players have already acquired a plethora of technical and tactical knowledge from their academy experience, and coach feedback enables them to customise their in-game performance.

Personal mindset was the second most important factor identified within the player development pathway. Soccer players need strong mental toughness to preserve their chances of moving up to a higher level of play because the game is unpredictable and unstable.^[15]^ Players with a growth mindset are generally dedicated and able to develop their skills through practice and hard work; they consider failures as teaching moments, are receptive to criticism and welcome new challenges for continuous self-improvement.^[16]^ The current study showed that community-based team players had significantly more positive personal mindsets than their school academy counterparts. The fact that academy players were not living with their families may have contributed to their pessimistic outlook and inability to handle stress. The results suggest that school academy players should be supported to develop a strong psychological mindset and coping mechanisms to assist them during difficult times, so they can manage a range of scenarios and still meet the pressure to succeed.^[11]^

The social support network was highlighted as the third crucial factor in the player development pathway. This correlates with previous research,^[17]^ which showed that social support, especially from family and peers, plays an important role in a player’s talent development trajectory. Early studies ^[4,16]^ found that family support, such as financial and time commitments and emotional support during game attendance, is vital to athlete development. In this study, community team players recorded significantly higher scores on the social support network than school academy players. This may be because academy players reside on school property for training. In contrast, community-based team players live at home. They may have access to a wider support network that includes parents, friends, coaches, siblings and others who can facilitate regular communication, performance feedback and goal-setting.

The sports psychology factor recorded the lowest score compared to the other player development factors. This finding supports Cooper^[8]^ who reported that among the factors influencing soccer players’ development, sport psychology had the lowest score with several players reporting an inability to handle pressure and a strong sense of being under pressure. The current data may suggest that coaches lack the necessary training to implement ‘sport psychology’ to assist their players in managing pressure and stress.^[18]^ School academy players gave this component a significantly lower value than their community-based team players, pointing to a glaring issue with academy soccer and supporting earlier findings that the academy setting lacks the essential resources to provide sufficient psychological support to players within the system.^[19]^ The current research suggests that coaches should receive sport psychology education to enhance players’ performance and help them cope with pressure.

### Limitations and future research

This study has a few limitations to consider when interpreting the results. First, the sample size was small, and the findings cannot be generalised to the South African population. Second, data was collected at one specific time, and causal relationships could not be established. Lastly, the study did not account for any confounding variables that might have affected how the results were interpreted, such as socioeconomic level, coaching quality and training experience. Therefore, to gather rich data and in-depth information about player development in the South African context, future research should use a larger sample size, include various factors not included in this study, and use other research measures such as qualitative designs (semi-structured or focus group interviews).

## Conclusion

The findings highlighted that coaching, personal mindset and social support networks are the most important factors influencing a player’s development trajectory. The results indicate that coaches should concentrate on players’ technical and tactical skills and provide regular feedback on their training to support their overall growth. Soccer coaches should foster support systems such as parents, siblings and peers to enhance players’ talent development trajectory. Furthermore, sport psychology was found to have the least impact on players’ growth. This finding demonstrates that more research needs to be done to assess how well coaches are equipped to provide psychological support. Coaching intervention strategies targeted at players, especially those in the academy, should be established to build a strong psychological mindset and coping mechanisms to help them through challenging times. This will enable them to handle various situations and still meet the pressure to succeed in the face of adversity.

## Figures and Tables

**Table 1:** Player development pathway between community and school academy players

Subscales (AU)	Community team n=51	School academy n=61	All n=112	p-value	Effect Size

Coaching	4.56±0.63	4.03±0.77	4.27±0.75	0.0001*	0.75
Sport science/performance analysis	4.28±3.57	4.01±0.63	3.89±0.83	0.0001*	0.10
Sport psychology	4.01±0.91	3.30±0.61	3.62±0.83	0.0001*	0.92
Social support network	4.36±0.57	4.01±0.63	4.17±0.63	0.003*	0.58
Personal mindset	4.60±0.48	3.94±0.58	4.24±0.63	0.0001*	1.24

* indicates p<0.05.

AU, arbitrary units.

**Table 2:** Player development pathway between YDP and PDP players

Subscales (AU)	Youth development phase n=73	Professional development phase n=39	p-value	Effect Size

Coaching	4.32±0.70	4.18±0.84	0.37	0.18
Sport science/performance analysis	3.87±0.85	3.94±0.82	0.71	0.08
Sport psychology	3.60±0.91	3.66±0.68	0.71	0.07
Social support network	4.16±0.66	4.20±0.56	0.72	0.06
Personal mindset	4.26±0.61	4.18±0.67	0.52	0.12

* indicates p<0.05.

YDP, Youth Development Phase; PDP, Professional Development Phase

## References

[1] Webb T, Dicks M, Brown DJ, O’Gorman J (2020). An exploration of young professional football players’ perceptions of the talent development process in England. Sport Manag Rev.

[2] (2011). Premier League. Elite Player Performance Plan.

[3] Bloom BS, Bloom BS (1985). Generalizations about Talent Development. Developing Talent in Young People.

[4] Côté J (1999). The influence of the family in the development of talent in sport. Sport Psychol.

[5] South African Football Association (2011). SAFA Regulations on Football Academies, Johannesburg.

[6] Ma TL, Simpkins S, Puente K (2021). Latinx and white adolescents’ reasons behind organized activity participation: The connections with cultural orientations, psychological engagement, and activity experiences. Appl Dev Sci.

[7] Diouf M, Miller J, Rothwell M (2024). Professional soccer players perceptions of the English Football Association 4 corner model in supporting first team transitions. J Sports Sci.

[8] Cooper A (2020). An investigation into the factors affecting player development within each phase of the academy pathway in English football academies. Soccer Soc.

[9] Harwood CG, Barker JB, Anderson R (2015). Psychosocial development in youth soccer players: Assessing the effectiveness of the 5Cs intervention program. Sport Psychol.

[10] Lagestad PA, Sæther SA, Ulvik A (2017). Differences in coaching feedback between coaches of junior elite soccer players and junior amateur soccer players. J Phys Educ Sport.

[11] Finn J, McKenna J (2010). Coping with academy-to-first-team transitions in elite English male team sports: The coaches’ perspective. Int J Sports Sci Coach.

[12] Taylor IM, Bruner MW (2012). The social environment and developmental experiences in elite youth soccer. Psychol Sport Exerc.

[13] Mills A, Butt J, Maynard I, Harwood C (2014). Toward an understanding of optimal development environments within elite English soccer academies. Sport Psychol.

[14] Van Yperen NW (2009). ‘Why some make it and others do not: Identifying psychological factors that predict career success in professional adult soccer’. Sport Psychol.

[15] Dweck CS (2009). Mindsets: Developing talent through a growth mindset. Olympic Coach.

[16] Fraser-Thomas JL, Côté J, Deakin J (2005). Youth sport programs: An avenue to foster positive youth development. Phys Educ Sport Pedagogy.

[17] Kubayi A, Coopoo Y, Morris-Eyton H (2016). Coaches’ preferences for continuing coaching education in South Africa. J Hum Kinet.

[18] Cushion CJ, Armour KM, Jones RL (2006). Locating the coaching process in practice: models ‘for’ and ‘of’ coaching. Physical education and sport pedagogy.

